# Relationship between Breast Feeding and Obesity in Children with Low Birth Weight

**DOI:** 10.5812/ircmj.11120

**Published:** 2013-08-05

**Authors:** Mitra Zarrati, Farzad Shidfar, Maryam Moradof, Farinaz Nasiri Nejad, Hossein Keyvani, Mohsen Rezaei Hemami, Elham Razmpoosh

**Affiliations:** 1 Department of Nutrition and Biochemistry, Faculty of Health, Tehran University of Medical Sciences, Tehran, IR Iran; 2 Department of Exercise Physiology, Islamic Azad University, Tehran, IR Iran; 3 Department of Physiology, Medicine Faculty, Tehran University of Medical Sciences, Tehran, IR Iran; 4 Department of Virology, Medicine Faculty, Tehran University of Medical Sciences, Tehran, IR Iran; 5 Cardiovascular Research Center, Rajaee Hospital, Tehran University of Medical Sciences Tehran, IR Iran; 6 Department of Nutrition, Faculty of Health, Qazvin University of Medical Science, Qazvin, IR Iran

**Keywords:** Low Birth Weight, Abdominal Obesity, Breast Feeding, Iran

## Abstract

**Background:**

Breast feeding appears to play a role in determining obesity and abdominal obesity during childhood, specifically in children with a history of low birth weight.

**Objective:**

The purpose of this study is to investigate the relation of breast-feeding with either of abdominal obesity and obesity among Iranian school children.

**Materials and Methods:**

A total of 1184 students (625 girls and 559 boys), aged 10 to 13 years old, were selected from 112 governmental elementary schools in Iran. Height, weight, waist circumference and blood pressure were measured using standard instruments and a pretested standardized questionnaire was performed for compiling information about family economics and educational level, first–degree family history of obesity, history of breast feeding, food pattern and birth weight, as well.

**Results:**

13.68% (n = 160) of students had a history of low birth weight, and 26.41% of them had abdominal obesity. Of all participants, 22.04% were overweight and 5.32% were obese which was more prevalent in girls than in boys (P = 0.03). First-degree family history of obesity (P = 0.001), excessive gestational weight gain (P = 0.001) and birth weight (P = 0.01) were significantly correlated with the prevalence of obesity and abdominal obesity during childhood. Moreover the prevalence of abdominal obesity in children with low birth weight was significantly correlated with breast feeding (P = 0.04); But this relation was not significantly about obesity in our participants (P = 0.9). Furthermore duration of breast feeding was significantly and inversely correlated with obesity and abdominal obesity in schoolchildren with low birth weight (P = 0.01).

**Conclusions:**

The results suggest that Breast feeding and its long-term consequences were important factors for preventing metabolic syndrome criteria in childhood and later years of life span. With regard to the increasing prevalence of obesity in children, more research is urgently needed to clarify whether breast feeding have negative consequences for the risk of chronic disease in children, especially in children with low birth weight.

## 1. Background

In many countries, childhood overweight and obesity have extended epidemic proportions (1). Moreover, many complications of obesity such as hypertension and chronic disease are seen in children nowadays (2). Recently endeavors made to understand the risk factors for overweight and obesity; in this regard breast feeding plays an important role. The short term benefits of breast feeding. It provides many health, economical, nutritional and emotional benefits to children as well as improving child growth (3). Increasing evidence (4) demonstrate that, having been breast fed may also have longer term benefits, including prevention of obesity especially before the pubertal onset (5, 6). As a result breast feeding and its duration play significant roles in prevention of childhood obesity. In children with low birth weight, consumption of supplement formula is more common, however researchers have shown that, prolonged breast feeding can reduce the risk of obesity during childhood. It might be related to some components exist in breast milk such as Grelin, Adipokines and Resistin which influence food intake regulation and lead to balance in energy intake during infancy (7).

Other factors that influence on childhood obesity are first degree family history of obesity, family economic and educational level, child meal patterns, duration of sleeping, physical activity level and birth weight (8, 9). On the other hand biological factors, genetics, environmental and socio-economic parameters affect and control the obesity from the early years of life to adulthood (10). Scientists have found that, not only high birth weight children, but also children with low birth weight (LBW), will be obese in their later life (11, 12). Children with LBW will gain weight more quickly which is called catch up growth, in order to make up their lack of growth. Thus high rapid weight gain during childhood may lead to an increase in abdominal obesity, insulin resistance and glucose intolerance (13).

Hales and Barker have demonstrated that low birth weight, which is a reflection of nutritional deprivation in uterus (14), might impair the development of the fetal pancreas, (15) and could lead to a predisposition to childhood obesity and non-communicable disease. They have also proposed that, obesity is mainly the result of environmental factors and genetic factors play little or no role in its progress, which is called the "thrifty phenotype" hypothesis (16). Furthermore high rapid weight gain, results in adipogenesis in children with LBW. Align with this, many studies confirmed that children with LBW have more body fat percentage than children with normal birth weight (NBW). Their catch up growth period might cause synthesis of visceral adipose tissue in their body (11) as well as fat accumulation that especially cause abdominal obesity.

## 2. Objectives

The present study attempts to investigate the association of breast feeding and its duration, with obesity and abdominal obesity among elementary school children who had a history of LBW.

## 3. Materials and Methods

A total of 1184 primary school students (625 girls and 550 boys), aged 10 to 13 years old, were selected randomly from 20 governmental elementary schools between 2011 and 2012 in Tehran, Iran. A standardized and validated questionnaire was supplied to mothers for collecting information about family economics and educational level, first degree family history of obesity, children’s food pattern, history and duration of breast feeding and birth weight of participants. Information on birth weight of children was collected retrospectively based on newborn demographic data. Ethical approval was provided by the committee of ethics in Tehran University of medical science.

Exclusion criteria included cardio vascular disease, diabetes mellitus, influenza, renal disease, dental infection and consumption of any drugs and supplements. To validate the questionnaire, it was administered to nutritionist and to evaluate the reliability, it was distributed to 20 schoolchildren who were not participated in the study. Weight was measured using digital scale, without shoes and heavy clothing with a precision of 0.1 Kg and height was measured without shoes to the nearest tenth of a centimeter using a portable stadiometer (Seca model 207 Germany). Likewise waist circumference was measured over skin by an inelastic plastic tape. Abdominal obesity status was classified as a waist circumference at or above 85th percentile of age-and-gender matched children from the National Health and Nutrition Examination survey (NHNES) III cohort (17). Overweight in children was defined as a BMI at or above 85th percentile and lower than the 95th percentile for the children of the same sex and age. Obesity was defined as a BMI above 95th percentile for the children of the same age and sex as well (18). BMI was calculated by dividing weight (Kg) by height square (m²). Low birth weight has been defined by the World Health Organization (WHO) as weight at birth of less than 2500 grams (19).

### 3.1. Statistical Analyses

Quantitative variables are presented as mean ± SD, relative frequency and absolute frequency. SPSS software (version 18.0) was used for statistical analyses. Differences between groups’ means were assessed by T-test, and Chi-square was used for comparing proportions. P-value ≤ 0.05 considered as statistically significant.

## 4. Results

1184 students, (625 girls and 559 boys) aged 10–13 years, were selected from 112 elementary school in, Tehran, Iran. 13.68% (n = 160) of participates were LBW, 26.41% (n = 309) were macrosom (more than 4000 gr) and 59.91% (n = 701) had normal weight at the time of birth. 5.32% of participants were obese and 22.04% of them had over weight which were both more common in girls than boys (P = 0.03). 25.34% of participants had abdominal obesity (ABO) that was more prevalent in boys compared with girls (26.48 % vs. 24.32%, P = 0.002). Our observation showed that, students whose mothers had bachelor's degree or higher were less obese and overweight, although the relationship was not significant (P > 0.05). We also found that, there was a significant relation between father's educational level (Masters of Science and higher degree) with obesity and ABO in students (P = 0.02). In other words, higher education of participants’ fathers was associated with a lower incidence of obesity and ABO. In our study more than half of the fathers and mothers were self-employed and house wives respectively. Our data demonstrated that fathers who were employed and mothers who work outside their homes, had obese children compared to others (P = 0.03). As it is indicated in Table 1, birth weight had a significant relationship with BMI, weight, waist circumference and systolic blood pressure (SBP) (P < 0.05) but not with other parameters, like height and diastolic blood pressure (DBP) (P > 0.05).

**Table 1.** Anthropometric Indicators, Systolic Blood Pressure (SBP) and Diastolic Blood Pressure (DBP) in Participates Based on Birth Weight

	Birth Weight (Mean ± SD^a^)			P-value^b^	Post Hoc^c^
	Less than 2500 Grams	2500-4000 Grams	More than 4000 Grams		
**BMI^a^ (Kg/m2)**	21.91 ± 5.26	18.78 ± 3.44	19.48 ± 3.61	0.029	1vs2, 2vs3, 1vs3
**Weight (Kg)**	47.23 ± 14.02	40.08 ± 9.49	42.87 ± 10.17	0.012	1vs2, 2vs3, 1vs3
**Height (cm)**	145.93 ± 7.12	145.49 ± 6.99	147.72 ± 7.10	0.341	1vs3 2vs3
**Waist Circumference (cm)**	77.08 ± 13.33	69.63 ± 9.63	71.21 ± 9.85	0.034	1vs2, 2vs3, 1vs3
**SBP (mmHg)^a^(mmHg)**	106.33 ± 16.14	100.46 ± 13.15	101.56 ± 12.87	0.037	1vs2, 2vs3, 1vs3
**DBP (mmHg)^a^(mmHg)**	70.56 ± 15.23	67.44 ± 10.40	67.48 ± 10.01	0.197	1vs2, 2vs3, 1vs3

^a^ Abbreviations: BMI, Body Mass Index; DBP: Diastolic Blood Pressure; SBP, Systolic Blood Pressure; SD, Standard Deviation;

^b^ ANOVA

^c^Based on post hoc analysis means not sharing a common letter are significantly different (P < 0.05). 1: Low Birth Weight group; 2: Normal Birth Weight group; 3: Macrosome group

Based on data demonstrated in Table 2 which illustrates the univariate analyses of predictor variables of obesity and ABO, determined that among participants, only first degree family history of obesity, excessive gestational weight gain and birth weight had significant relation with the prevalence of obesity and ABO (P < 0.05). In addition, breast feeding and its duration had significant relation with obesity (P = 0.03 and P = 0.04 respectively); but they had no meaningful effect on ABO.

**Table 2.** The Relationship between Predictor Variables with Obesity and Abdominal Obesity in Iranian Children

	Obesity				Abdominal Obesity (ABO)			
	Normal, Frequency (Relative Frequency %)	Obesity, Frequency (Relative Frequency %)	Total, Frequency (Relative Frequency %)	P-value^a^	Normal, Frequency (Relative Frequency %)	ABO, Frequency (Relative Frequency %)	Total, Frequency (Relative Frequency %)	P value^a^
**Gender**				0.19				0.39
Girls	444 (51.63)	181 (55.86)	625 (52.79)		473 (53.51)	152 (50.67)	625 (52.79)	
Boys	416 (48.37)	143 (44.14)	559 (44.14)		411 (46.49)	148 (49.33)	432 (47.21)	
**First Degree Family Obesity**				0.001				0.001
Yes	257 (30.06)	175 (54.35)	432 (36.70)		274 (31.14)	158 (53.20)	432 (36.70)	
No	598 (69.94)	147 (45.65)	745 (63.30)		606 (68.86)	139 (46.80)	745 (63.30)	
**Severe Gain Weight in Pregnancy**				0.001				0.001
Yes	116 (13.49)	77 (23.91)	193 (16.33)		118 (13.35)	75 (25.17)	193 (16.33)	
No	743 (86.40)	245 (76.09)	988 (83.59)		766 (86.55)	223 (74.83)	988 (83.59)	
**BreastFeeding**				0.03				0.74
Yes	791 (92.08)	293 (90.71)	1084 (91.71)		812 (92.06)	272 (90.67)	1084 (91.71)	
No	65 (7.57)	29 (8.98)	94 (7.95)		70 (7.63)	27 (9.00)	94 (7.95)	
**Duration of Breast Feeding**				0.04				0.74
Less than 6 months	109 (13.46)	46 (15.33)	155 (13.96)		113 (13.58)	42 (15.11)	155 (13.96)	
6-12 months	135 (16.67)	44 (14.67)	179 (16.13)		137 (16.47)	42 (15.11)	179 (16.13)	
12-24 months	566 (69.88)	210 (70.00)	776 (69.91)		582 (69.95)	194 (69.78)	776 (69.91)	
**Birth Weight**				0.001				0.001
< 2500gr	69 (8.11)	91 (28.53)	160 (13.68)		80 (9.15)	80 (27.03)	160 (13.68)	
2500-4000 gr	555 (65.22)	146 (45.77)	701 (59.91)		559 (63.96)	142 (47.97)	701 (59.91)	
> 4000gr	227 (26.67)	82 (25.71)	309 26.41		235 (26.89)	74 (25.00)	309 (26.41)	

^a^ Chi Square Test

54.35% of obese and 53.20% of ABO participants had first degree family history of obesity (P = 0.001) (Table 2), which is more prevalent in girls (40%) than in boys (33.03%).

**Figure 1.**
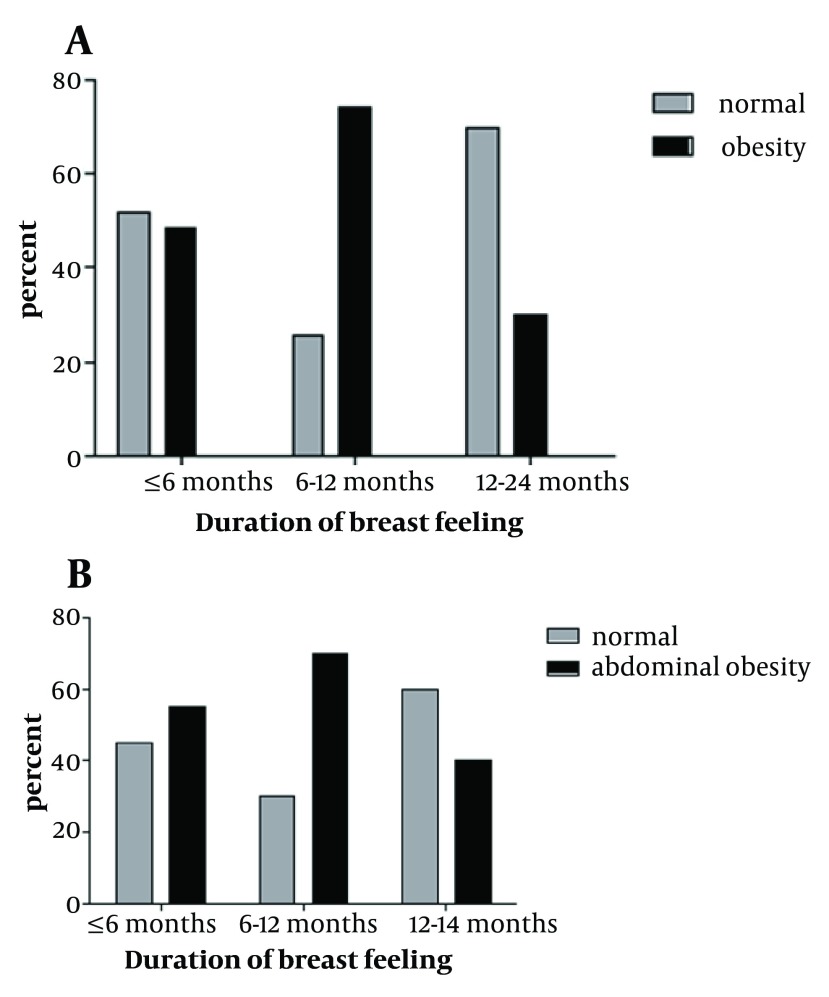
Distribution of BMI (A) and abdominal Obesity (B) by Breast Feeding in Children with Low Birth Weight. * P < 0.05; ** P > 0.05; BMI: Body Mass Index

28.53% and 27.03% of the children with LBW were obese and had ABO respectively. Among LBW children, normal birth weight and macrosom children, history of LBW, was statistically and significantly more effective on childhood obesity and ABO (P < 0.05). Figure 1 shows that, breast feeding had significant relationship with abdominal obesity (P = 0.01). But there was no significant relation between breast feeding and obesity in participates with low birth weight (P = 0.9).

**Figure 2.**
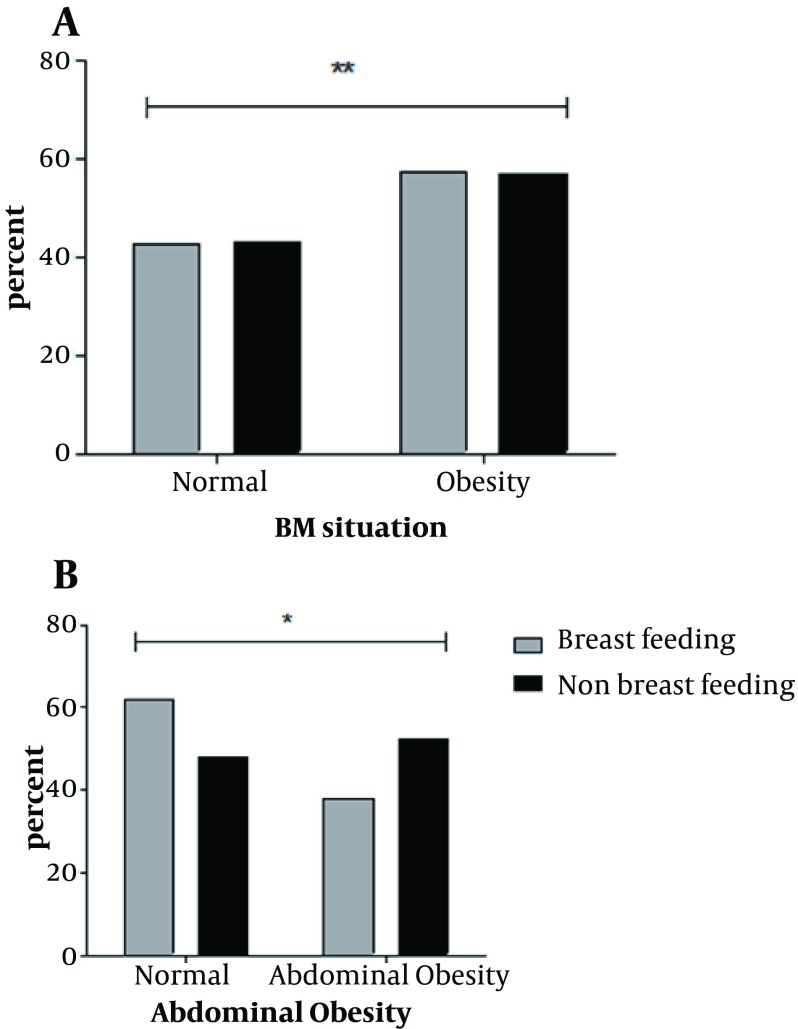
Distribution of Obesity (A) and Abdominal Obesity (B) by Duration of Breast Feeding in Children with Low Birth Weight

Our data reported that duration of breast feeding had significant relationship with childhood obesity and abdominal obesity in LBW group (P = 0.03 and P = 0.002 respectively). Students with low birth weight, who had been breast fed for 1 to 2 years, showed lower percent of obesity and ABO during childhood (Figure 2).

## 5. Discussions

High rapid weight gain occurs in LBW children during the first year of their life which might result in later obesity, chronic diseases and early mortality in adulthood. On the other hand, high blood pressure and insulin resistance is also more common in children with a history of LBW (20). Although it is obvious that obesity prevalence varies across populations (21), our findings indicated that, childhood obesity and abdominal obesity are nutritional problems in Iran. Moreover, as there are different cut-off values and references for defining obesity and also different age groups in studies, it is difficult to compare the rate of childhood obesity in various populations. As a result, there is a hypothesis named “thrifty phenotype” which explain the process of change and happen during fetal age and, thus might lead to abnormalities in later life.

Menstrual periods in women during resulted in selection pressures in favor of a thrifty genotype that leads to highly efficient fat storage during periods of abundance (22). In the current climate of food overabundance and sedentary lifestyle, this thrifty genotype suggested to be the predictor of disadvantageous metabolic phenotypes. The hypothesis inferred that, adaptation to fetal nutrient-conserving that occurs in response of intrauterine malnutrition, will be overcome by postnatal nutrient abundance, although it could be the cause of chronic diseases and metabolic syndrome in later life (23).

The trend of fetus growth in uterus has been demonstrated for no more than a decade. Furthermore it was observed that, birth weight can be effective on the incidence of chronic problems such as obesity and accumulation of abdominal fat during childhood ( 20 ). The present study suggests that obesity and abdominal obesity were more prevalent in children with a history of LBW compared with other participants. Meanwhile, our study demonstrated that some other involving factors such as first degree family history of obesity, excessive gestational weight gain, duration and history of breast-feeding could be effective on obesity and ABO during childhood, as well (Table 2). Children with ABO and without general obesity, have high level of Leptin and Visfatin and low level of Adiponectin which are important risk factors for metabolic syndrome in children ( 24 ).

In the present study, a statistical significant difference in abdominal obesity was seen in breast-fed children compared with formula- or mixed-fed groups. Likewise, we found that, breast-feeding for six months or more was a protective factor against obesity and abdominal obesity in elementary school children which was in accordance with those of Scott and coworkers that was performed in Australia (25). According to our results, breast feeding and particularly its duration, could be effective on obesity and ABO during childhood. Manco et al. (23) conducted a study in Italy which showed that 8 to 9 year-old children who have been breast-fed had significantly greater insulin sensitivity and lower insulin secretion level in comparison with formula-fed group. One of the unique advantages of Manco study was blood sampling assessment, which was not performed in our study. Abdominal fat accumulation is significant in LBW children, due to high level of serum leptin and thus increased insulin resistance which consequently leads to diabetes mellitusr (26).

Present study demonstrated that low prevalence of obesity and ABO was seen in children who have been breat fed for 1 to 2 years. On the other hand, extended breastfeeding had an important impact on the prevention of chronic disease in children. Many investigations have reported the beneficial effects of history and duration of breast-feeding on various aspects of child health. However Vafa et al. (27) found no significant correlation between these parameters and BMI in children. In a meta-analysis study, conducted by Owen et al. (28), the effect of breast feeding on average BMI was assayed and it was showed that mean BMI was slightly lower among breast-fed subjects, although adjusting for socioeconomic status, maternal BMI, and maternal smoking eliminate the effect.

Data shown in Table 2 demonstrates the relation between ABO and birth weight and it is in accordance with those of Barker, which was performed among 216 white, girls aged 14 to 16 years old and indicated that low birth weight was an important predictor factor of the prevalence of abdominal obesity in children. Subjects with LBW followed by catch-up-growth in early years of their life had high level of growth hormone during childhood and were more susceptible to abdominal obesity than other groups. In early childhood, obesity and ABO result in insulin resistance and consequently type 2 diabetes mellitus ( 29 - 31 ). It has been established that the prevalence of obesity was more among girls with a history of LBW compared with boys which is similar to our results. Align with these findings; these girls might be at risk of having uterine abnormalities and infertility in their later life. Hence these children need more attention from their birth time and particularly during their childhood ( 32 ).

However our results were not in accordance with those of Peter S that was conducted in Hungary on 1334 subjects aged 7 – 19 years old. They reported that the prevalence of obesity was higher among participants with a history of macrosomia than LBW subjects. One might argue that this study contains different age groups comparing to our study, while hormonal alterations especially with genetic attitude could effect on obesity during maturity (33). Moreover one of the unique advantages of the study conducted by Peter and coworkers was measuring the body fat percentage of all participants which was not performed in our study. On the other hand the result of our study was inconsistent with those of Hirchler et al. (34), performed in Buenos among low level socioeconomic status regions. They found no significant relationship between LBW and childhood obesity. On the other hand, no statistical prevalence of obesity and metabolic syndrome was observed among LBW and normal body weight children. By contrast, subjects with high birth weight (HBW), had some components of metabolic syndrome and other which leads to obesity and overweight.

Our study indicated that the average body mass index (BMI) of macrosome subjects was lower than the average body mass index (BMI) of students with LBW (Table 1). On the other hand it was observed that high birth weight was not a risk factor for obesity incidence in early years of life, since other effective factors such as gestational weight gain could affect the outcome. One of the advantages of our study was considering gestational weight gain which was not mentioned in Hirchler 's study. The present study reported that excessive gestational weight gain might cause obesity in offspring which is an independent predictor factor for childhood obesity. We also investigated the association of parental occupational level with either of childhood ABO and obesity. Mothers with high level of education had beneficial knowledge for controlling nutrition pattern and food intake of their children. In addition high level of parental education was significantly inversely associated with BMI in children. The Study of Hirchler ( 34 ) was just restricted to low socioeconomic status regions and hence the outcome showed that LBW children had low BMI in the next years of life.

It has been proved that lower parental educational level led to lower knowledge and hence lower control of food pattern over their children (35). Factors indicating the more prevalence of obesity in high birth weight schoolchildren consist of increasing the amount of fat cells (hypertrophic obesity) in their body and thus more fat accumulation. Though, breast feeding can even diminish the probability of obesity in these children in their later years of life. Meanwhile as history and duration of breast-feeding were not mentioned in Hirchler study, it is not possible to discuss the exact reason of later obesity in high birth weight schoolchildren.

In addition in the CASPIAN study performed by Kelishadi (36) among 21111 students in three different education levels showed that the percentage of obesity and overweight prevalence in Iran was higher in boys than girls, which was in conflict with our results. the large sample size in mentioned study might cause this confliction. However they found that the percentage of ABO in Iran was higher among boys than girls which were in line with our results. Many studies proved that breast feeding and the length of that have beneficial effect on preventing obesity and non-communicable disease in childhood and adulthood; thus recommending mothers to breast fed their children for at least 6 – 12 months, could have important influence on preventing any of them among children. First degree family history of obesity, excessive gestational weight gain and birth weight had significant relationship with the prevalence of obesity and abdominal obesity during childhood. The prevalence of abdominal obesity in students with low birth weight was significantly correlated with breast feeding. In addition as it has been proved, special consideration to LBW newborn children during the process of their growth is necessary. However the trend of fetus growth in uterus has been proved for no more than a decade and more researches is necessary for corroborating the role of LBW on the prevalence of obesity in the next years of life. Furthermore some factors like obesity first degree family history of obesity and excessive gestational weight gain in our study has been found as impressive parameters on obesity and abdominal obesity.
